# Health and Working Beyond Retirement Age: Exploring Racial and Gender Intersectionality

**DOI:** 10.1093/geroni/igab046.2182

**Published:** 2021-12-17

**Authors:** Ronica Rooks, Allison Leanage

**Affiliations:** 1 University of Colorado Denver, Denver, Colorado, United States; 2 McMaster University, Hamilton, Ontario, Canada

## Abstract

Little longitudinal research exists on health and working among older racial and ethnic minority adults. Following previous cross-sectional research, we examine the Health, Aging, and Body Composition (HABC) study comparing working vs. not working overtime among older adults. We hypothesize: 1) Black vs. White adults are more likely to work; 2) Black vs. White differences in working are greater among women than men; and 3) Working relates to fewer prevalent health problems than not working. We used gender-stratified descriptive statistics and generalized mixed-effects logistic regression with covariate adjustments to analyze the HABC cohort study, with community-dwelling, well-functioning Black (42%) and White older adults aged 70-79 in year 1 (n=3,069) to year 6 (n=2,091). We found support for all three hypotheses. Black vs. White adults were more likely to work overtime. Women were less likely to work overtime compared to men. White women were less likely to keep working compared to men and Black women. Lastly, older adults with fewer chronic conditions were more likely to continue working. Our study finds racial and gender differences among older adults working overtime. Intersectionality plays a role in older adults’ health and work disparities, leading us to explore the needs and/or benefits of working past retirement in specific groups. Our policy implication is for society to pro-actively invest in older adults’ health and productive activities, which may act as social determinants of health solutions to reduce disparities and growing social safety net program costs.

